# Reduction of the Cholesterol Sensor SCAP in the Brains of Mice Causes Impaired Synaptic Transmission and Altered Cognitive Function

**DOI:** 10.1371/journal.pbio.1001532

**Published:** 2013-04-09

**Authors:** Ryo Suzuki, Heather A. Ferris, Melissa J. Chee, Eleftheria Maratos-Flier, C. Ronald Kahn

**Affiliations:** 1 Joslin Diabetes Center, Harvard Medical School, Boston, Massachusetts, United States of America; 2 Beth Israel Deaconess Medical Center, Harvard Medical School, Boston, Massachusetts, United States of America; University of Cambridge, United Kingdom

## Abstract

The sterol sensor SCAP is a key regulator of SREBP-2, the major transcription factor controlling cholesterol synthesis. Recently, we showed that there is a global down-regulation of cholesterol synthetic genes, as well as SREBP-2, in the brains of diabetic mice, leading to a reduction of cholesterol synthesis. We now show that in mouse models of type 1 and type 2 diabetes, this is, in part, the result of a decrease of SCAP. Homozygous disruption of the *Scap* gene in the brains of mice causes perinatal lethality associated with microcephaly and gliosis. Mice with haploinsufficiency of *Scap* in the brain show a 60% reduction of SCAP protein and ∼30% reduction in brain cholesterol synthesis, similar to what is observed in diabetic mice. This results in impaired synaptic transmission, as measured by decreased paired pulse facilitation and long-term potentiation, and is associated with behavioral and cognitive changes. Thus, reduction of SCAP and the consequent suppression of cholesterol synthesis in the brain may play an important role in the increased rates of cognitive decline and Alzheimer disease observed in diabetic states.

## Abstract 2

A conditional knockout of SCAP in the mouse brain identifies impaired cholesterol synthesis as a potential link between diabetes and altered brain function.

## Author Summary

Diabetes is associated with an increased risk of Alzheimer disease, depression, and cognitive decline, but the causal link underlying these associations is unclear. We previously showed that in diabetic mice there is a reduction in brain synthesis of cholesterol, which is required for normal formation of synapses between neurons. Here we show that this deficit is caused, in part, by a reduction in the levels of SCAP, a protein known to help regulate cholesterol synthesis by promoting the relocalization, cleavage, and liberation of the key transcription factor SREBP2. These changes in cholesterol biosynthesis are rescued by treatment of the diabetic mice with insulin. When the level of SCAP in the brains of non-diabetic mice is lowered by genetic manipulation, there is a decrease in cholesterol synthesis in the brain, and this results in impaired signaling between neurons, memory deficits, and abnormal responses to stress. These findings indicate that the reduction in SCAP associated with diabetes can contribute to changes in cognitive function in this disease.

## Introduction

The brain is the most cholesterol rich organ in the body, containing more than 20% of the sterol pool and almost all of the cholesterol is produced in situ [1]. Multiple in vitro studies have indicated that cholesterol in the brain is important for synapse biogenesis and vesicle formation [2],[3]. Cholesterol synthesis is a highly regulated process controlled by the master transcriptional regulator SREBP-2. SREBP-2 is transcribed and translated into an inactive precursor that is sequestered in the endoplasmic reticulum (ER). However, when sterol levels are low, the sterol sensor SCAP is able to chaperone SREBP-2 to the Golgi apparatus where it is cleaved to release a transcriptionally active form that can enter the nucleus [4]. Conversely, in times of sterol abundance, SCAP is bound by sterols and remains sequestered in the ER along with the unprocessed SREBP-2 [4].

Diabetes mellitus is a multifactorial disease due to deficient insulin secretion and/or action, resulting in hyperglycemia, alterations in lipid metabolism, and a variety of complications in tissues throughout the body. These complications extend to the central nervous system (CNS), including cognitive dysfunction and behavioral changes, and are observed both in type 1 and type 2 diabetes [5]. Studies have shown altered information processing, psychomotor efficiency, attention, and mental flexibility in type 1 diabetes, whereas type 2 diabetes more often affects memory, psychomotor speed, and executive function [6]. In addition, changes have been observed on imaging of the hippocampi of both type 1 and type 2 diabetics [7],[8].

We recently demonstrated that in mouse models of diabetes there is a broad reduction in the expression of genes in the cholesterol biosynthetic pathway throughout the brain, resulting in decreased brain cholesterol synthesis [9]. This is accompanied by decreased expression of SREBP-2, which is more pronounced at the protein than the mRNA level [9]. Simultaneous reduction in cholesterol content and cholesterol biosynthesis suggests that, in the brain, diabetes causes a defect at the level of the sterol-sensing molecules, which constitute a negative-feedback system on SREBP-2 processing and maintenance of cellular cholesterol content. Here we show that the sterol sensing protein SCAP, which plays a key role in the post-transcriptional regulation of SREBP-2, is decreased in the brains of diabetic mice. Brain-specific heterozygous SCAP knockout mice have reduced levels of cholesterol genes and exhibit a 30% reduction in brain cholesterol synthesis, mimicking what is observed in diabetes. This results in defects at the electrophysiological and cognitive level. Thus, diabetes results in a reduction of SCAP expression, which contributes to a reduction of brain cholesterol synthesis. Reduction in SCAP leads to an impairment in neuronal transmission and cognitive dysfunction that may contribute to the neurological complications observed in diabetic states.

## Results

### SCAP Protein Is Reduced in the Brains of Diabetic Mice

**Figure 1:**
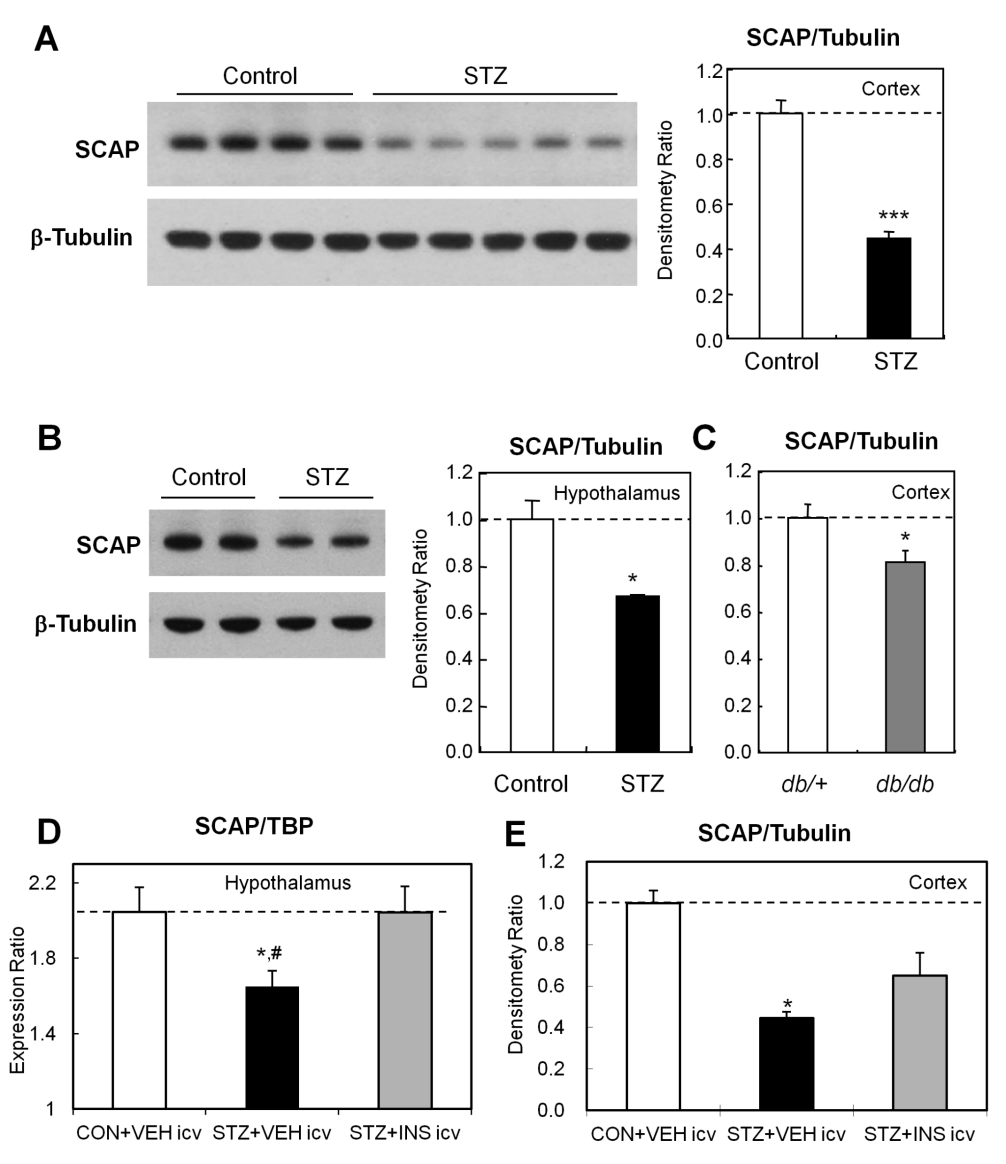
Diabetes reduces SCAP in the brain and is rescued by ICV insulin. Densitometry of the Western blots for SCAP of cytoplasmic extracts were normalized by the level of β-tubulin as an internal control. (A) Cortex of control and STZ-induced diabetic mice. (B) Hypothalami of control and STZ-induced diabetic mice. (C) Cortex of *db/+* and *db/db* mice. (D) *Scap* mRNA expression levels in the hypothalami of control, STZ-induced diabetic, and STZ-induced diabetic mice treated with insulin. (E) Western blots of SCAP of cytoplasmic extracts, normalized to β-tubulin from the cortex of control, STZ-induced diabetic, and STZ-induced diabetic treated with ICV insulin, *, *p*<0.05; ***, *p*<0.001; #, *p*<0.05 compared to ICV insulin group, by *t*-test.

Recently we demonstrated that diabetes produces a global suppression of the enzymes of cholesterol biosynthesis and their master transcriptional regulator SREBP-2 in the brain; this results in reduced cholesterol biosynthesis and altered ability of neurons to form synapses and synaptic vesicles [9]. Since reduction of cholesterol and its precursors would normally drive a compensatory increase in SREBP-2 and cholesterol biosynthesis, we speculated that sterol-sensing might be impaired in the brains of diabetic mice. SCAP, a binding partner and a chaperone protein of SREBP-2, plays an essential role in sterol feedback regulation, serving as the major sterol-sensing molecule [4]. Western blotting of extracts of brains from streptozotocin (STZ)-treated mice (a model of type 1 diabetes) revealed an ∼50% decrease in SCAP protein in the mouse cerebral cortex (Figure 1A). This reduction of SCAP protein in the STZ diabetic mouse was not limited to the cortex and was observed throughout the brain, with a 33% decrease in the hypothalamus (Figure 1B) and a 31% decrease in the thalamus (unpublished data). There is also a significant, but smaller, reduction of SCAP protein in the cerebral cortices of *db/db* mice (a model of type 2 diabetes), consistent with a modest down-regulation of genes of the cholesterol synthesis pathway in the brains of these animals (Figure 1C). The decrease in *Scap* mRNA in STZ diabetic mice was completely rescued by three injections of insulin into the intracerebroventricular (ICV) space over the course of 30 h (Figure 1D). ICV insulin also resulted in a partial rescue of SCAP protein during this short treatment period (Figure 1E). This occurred with no change in blood glucose levels [9]. By contrast, there were no changes in *Scap* message after treatment of leptin deficient ob/ob mice with leptin (unpublished data).

### Homozygous Deletion of the SCAP gene in the Brain Causes Perinatal Lethality, Decreased Cholesterol Content, Microcephaly, and Gliosis

**Figure 2:**
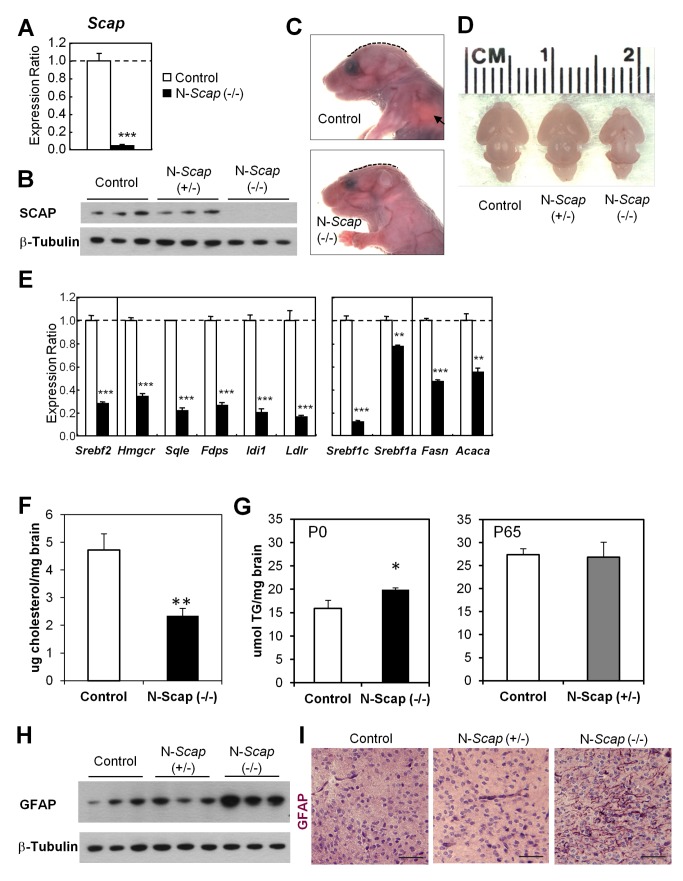
CNS-specific homozygous *Scap* deficient (N-*Scap* [−/−]) mice exhibit microcephaly and gliosis. (A) *Scap* mRNA expression levels in the brains of P0 mice by qPCR, average expression values in Control assigned a value of 1. Bars denote mean ± SEM. (B) Western blots of cytoplasmic extracts from brains of control, N-*Scap* (+/−), and N-*Scap* (−/−) mice. (C) Appearance of control and N-*Scap* (−/−) neonates. Dashed lines indicate difference in curvature of the skulls. Aerated lungs were visible in the control pups (arrow), whereas N-*Scap* (−/−) pups did not start breathing and died soon after birth. (D) Appearances of the brains at E18.5. (E) Comparison of gene expression for the cholesterol synthesis (SREBP-2 pathway) and fatty acid synthesis (SREBP-1 pathway) in brains of Control and N-*Scap* (−/−) neonates. (F) Total brain cholesterol content normalized to tissue weight. (G) Total brain triglyceride content normalized to tissue weight in P0 and P65 mice. (H) Western blots of cytoplasmic extracts from brains of Control, N-*Scap* (+/−), and N-*Scap* (−/−) neonates for GFAP protein. (I) Immunohistochemistry for GFAP in P0 brains. Scale bar = 25 µm. *, *p*<0.05; **, *p*<0.01; ***, *p*<0.001 by *t*-test.

To determine the consequences of reduced SCAP content in the brain in the absence of diabetes, we created mice with homozygous and heterozygous loss of SCAP specifically in the brain by crossing mice carrying a modified *Scap* gene with LoxP sites surrounding exon 1 [10] with mice expressing Cre-recombinase under control of the rat nestin promoter and enhancer [11]. This results in inactivation of the *Scap* gene in both neuronal and glial cells beginning on day e11.5 [11]. Homozygous *Scap*-ablated embryos (*nestin-Cre^+/−^:Scap^lox/lox^*, designated as N-*Scap* [−/−]) were obtained at normal Mendelian frequency; however, all of the homozygous N-*Scap* (−/−) mice died immediately after birth on postnatal day 0 (P0). Analysis of brains of these mice revealed an almost complete (>95%) absence of *Scap* mRNA and SCAP protein (Figure 2A and 2B). The N-*Scap* (−/−) neonates exhibited almost normal gross morphology, but had flat skulls and uninflated lungs (Figure 2C). Likewise, when mice were delivered by Cesarean section on embryonic day 18.5 (E18.5), all of the control (*nestin-Cre^−/−^:Scap^lox/wt^* or *nestin-Cre^−/−^:Scap^lox/lox^*) and N-*Scap* (+/−) (*nestin-Cre^+/−^:Scap^lox/wt^*) littermates initiated breathing, whereas N-*Scap* (−/−) failed to do so. The flat skull in N-*Scap* (−/−) was due to microcephaly; brains from N-*Scap* (−/−) mice exhibited an overall reduction in size of 43% by weight compared with those from the control littermates (50±10 mg versus 87.5±15 mg in controls, *p* = 0.003) (Figure 2D). There were no gross morphologic changes in the brain other than the decrease in size.

Gene expression analysis of the brains of N-*Scap* (−/−) mice revealed a pattern consistent with a loss of SCAP activity. Thus, SREBP-2 (*Srebf2*) and its downstream genes (*Hmgcr*, *Sqle*, *Fdps*, *Idi1*, and *Ldlr*) were all markedly down-regulated by 70%–90% (Figure 2E). Expression of the SREBP-1c (*Srebf1c*) gene was also reduced by 88%, whereas expression of its downstream genes (*Fasn* and *Acaca*) were only decreased by half (Figure 2E). The reduction in cholesterol synthetic enzymes was accompanied by a 50% reduction in total cholesterol content per gram of brain tissue (2.3±0.6 versus 4.7±1.2 µg of cholesterol/mg of brain in controls, *p*<0.01) (Figure 2F). In contrast, total brain triglyceride content was increased in N-*Scap* (−/−) mice compared to controls (19.9±0.4 versus 15.9±1.7 µmol of triglycerides/mg of brain in controls, *p*<0.05) (Figure 2G). Measurement of triglycerides in adult N-*Scap* (+/−) brains showed no difference when compared to controls (26.8±3.3 versus 27.4±1.3 µmol of triglycerides/mg of brain in controls) (Figure 2G).

Homozygous deletion of the *Scap* gene in the brain also caused marked changes in brain histology and cell type distribution. Expression of glial fibrillary acidic protein (*Gfap*), an astrocyte marker, was up-regulated by more than 3-fold in the whole brain of N-*Scap* (−/−) mice, whereas the microglia marker F4/80 (*Emr1*) was reduced by 59% and the neuronal marker MAP-2 (*Matp2*) was unchanged (Figure S1). The increase in *Gfap* mRNA correlated with a significant increase in GFAP protein in the N-*Scap* (−/−) brain (Figure 2H), which was confirmed by immunohistochemistry showing a robust increase of GFAP- positive fibers in N-*Scap* (−/−) brains resulting in a histological picture resembling gliosis (Figure 2I).

### Haploinsufficiency of *Scap* in the Central Nervous System Suppresses the SREBP-2 Pathway, In Vivo Cholesterol Synthesis, and Synapse Formation in the Brain

**Figure 3:**
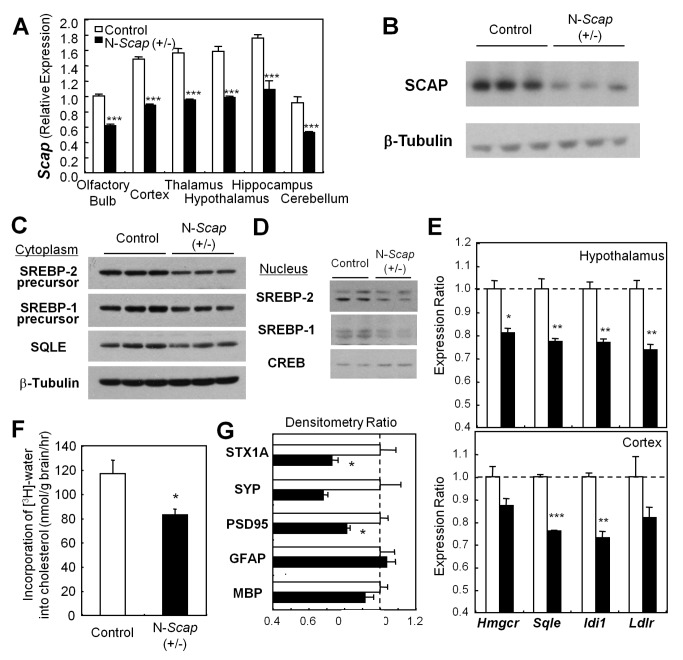
N-*Scap* (+/−) mice have suppressed cholesterol synthesis leading to a reduction of synapse markers. (A) *Scap* mRNA expression levels in brain regions of 11-wk-old control (open bars) and N-*Scap* (+/−) mice (closed bars) were measured by qPCR, and average expression values in olfactory bulb from Control littermates assigned a value of 1. (B) Western blots of whole cell extracts from cerebral cortices of 5-wk-old mice. (C) Protein levels of SREBP precursors and squalene epoxidase (SQLE) in cytoplasmic extracts from cerebral cortices. (D) Cleaved (transcriptionally active) SREBPs in nuclear extracts from cerebral cortices. CREB is used as an internal control. (E) mRNA expression levels of cholesterol-related genes in the hypothalami and cortices of 11-wk-old mice. (F) In vivo cholesterol synthesis in the whole cerebrums was assessed for Control and N-*Scap* (+/−) mice. (G) Densitometry of Western blots for synapse markers of cytoplasmic extracts from cerebral cortices of 5-wk-old mice. STX1A, syntaxin 1A; SYP, synaptophysin; PSD95, post-synapse density 95; GFAP, glial fibrillary acidic protein; MBP, myelin basic protein. Values are normalized to β-tubulin. *, *p*<0.05; **, *p*<0.01; ***, *p*<0.001 by *t*-test.

To better mimic the 50% reduction in SCAP in the brains of STZ-diabetic mice we characterized mice with haploinsufficiency of the *Scap* gene in the brain. qPCR analysis of *Scap* mRNA in extracts of brain regions dissected from N-*Scap* (+/−) mice revealed an approximately 40% reduction of *Scap* gene expression in all parts of the brain examined, including cerebral cortex and hypothalamus (Figure 3A). By Western blotting of extracts of the cortex, expression of SCAP protein was reduced slightly more (60%, when normalized with β-tubulin) in N-*Scap* (+/−) mice (Figure 3B).

Previous studies have shown that knockout of the *Scap* gene in the liver causes a reduction in the amounts of both the precursor and the nuclear forms of SREBP-2 and SREBP-1 [10]. We found a similar significant reduction of both SREBP-2 and SREBP-1 precursors in the cytoplasmic extract of cerebral cortices of N-*Scap* (+/−) mice (Figure 3C), as well as a reduction in the mature nuclear forms of these transcription factors (Figure 3D). This resulted in a decrease in the expression of genes downstream of SREBP-2 (*Hmgcr*, *Sqle*, *Idi1*, and *Ldlr*) by 10%–27% in the cerebral cortex and hypothalamus, as well as some decrease in *Srebf2* mRNA (Figure 3E). The reduction of squalene epoxidase at the protein level was also confirmed by Western blotting (Figure 3C). The decrease in SREBP-1 did not result in a decrease in expression of the downstream protein fatty acid synthase but did cause a 20% reduction in acetyl-CoA carboxylase in the N-*Scap* (+/−) mice. There were large decreases in these proteins in the N-*Scap* (−/−) mice (Figure S2). The reduction in cholesterol synthesis was not accompanied by a similar decrease in cholesterol catabolism. Levels of Cyp46a1 mRNA were the same in the cerebral cortices and hypothalami of control and N-*Scap* (+/−) mice, and whole brains of control and N-*Scap* (−/−) mice (Figure S3).

To determine if the down-regulation of cholesterologenic genes could affect in vivo cholesterol synthesis, we assessed cholesterol synthesis in the brains of N-*Scap* (+/−) mice in vivo using tritiated water. This revealed a 29% reduction in cholesterol synthesis in the brains (*p*<0.05) of N-*Scap* (+/−) mice (Figure 3F), closely paralleling the change observed in STZ-diabetic mice [9]. Synapse marker proteins, including syntaxin-1A (STX1A) and post-synapse density 95 (PSD95) were also decreased in the N-*Scap* (+/−) mouse cerebral cortices (Figure 3G); similar to what has been observed in STZ-diabetic mice [12]. This occurred with no change in glucose levels (after a 4-h fast 182±6.7 versus 175±8 mg/dl in controls) or fed insulin levels (0.32±0.02 versus 0.33±0.04 ng/ml in controls). Thus, the heterozygous N-*Scap* (+/−) mice mimic the reduction of SCAP protein, the impaired cholesterol biosynthesis, and the decrease in synapse markers observed in the brains of diabetic mice, but without the concomitant changes in glucose or insulin levels.

### Impaired Synaptic Transmission in the Hippocampus of N-*Scap* (+/−) Mice

**Figure 4:**
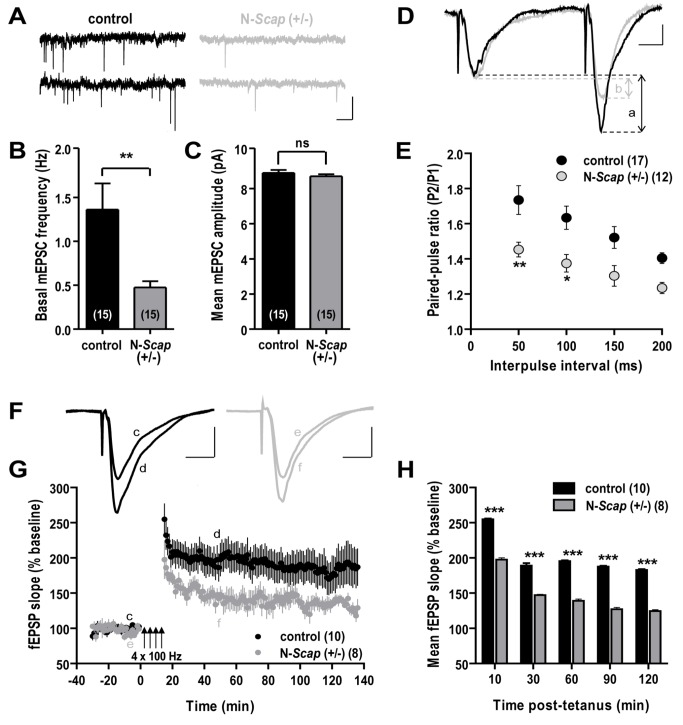
Synaptic transmission is impaired in N-*Scap* (+/−) mice. (A) Sample traces of mEPSC events at area CA1 neurons. Scale: 20 pA, 500 ms. (B) Basal frequency of mEPSCs at area CA1. (C) Mean amplitude of mEPSCs at area CA1 neurons. (D) Sample traces of a pair of fEPSPs elicited with an interpulse interval of 50 ms at the Schaeffer collateral pathway. The relative increase in fEPSP amplitude at the second pulse was calculated as the paired-pulse ratio (P2/P1) in control (a) and N-*Scap* (+/−) (b) hippocampal slices. Scale: 0.5 mV, 10 ms. (E) Paired-pulse ratios elicited following all interpulse intervals tested from hippocampal slices. (F) Sample traces of fEPSPs elicited pre- and post-tetanic stimulation (4×100 Hz trains) of CA1 synapses from control (c, d) and N-*Scap* (+/−) (e, f) hippocampal slices. Scale: 0.5 mV, 10 ms. (G) LTP, measured as the percent increase in fEPSP slope from baseline, achieved in slices from N-*Scap* (+/−) mice (gray) and controls (black). (H) LTP measured at 10, 30, 60, 90, and 120 min post-tetanus in CA1 synapses. *, *p*<0.05; **, *p*<0.01; ***, *p*<0.001; ns, not significant.

To determine whether changes in cholesterol metabolism induced by the decrease of brain SCAP protein might alter synaptic transmission, we performed intracellular and extracellular electrophysiological recordings from neurons in the area CA1 region of the hippocampus, an area of the brain with demonstrated abnormalities on imaging in humans with type 1 and type 2 diabetes [7],[8]. Intracellular recording of miniature excitatory postsynaptic current (mEPSC) events were monitored by continuous voltage-clamp to assess differences in frequency and amplitude of presynaptic events at CA1 neurons. The basal frequency of mEPSCs of area CA1 neurons was reduced by more than 60% in N-*Scap* (+/−) mice (0.5±0.1 Hz; *n* = 15 cells; *p*<0.01) compared to control mice (1.4±0.3 Hz; *n* = 15 cells) (Figure 4A and 4B), but no differences in mEPSC amplitudes were observed between the two genotypes (control: 8.9±0.2 pA; N-*Scap* (+/−): 8.7±0.1 pA; *n* = 1500 events) (Figure 4C). These results strongly implicate an effect of SCAP reduction on presynaptic neurotransmitter release, with no differences at the postsynaptic membrane.

To further assess the effect of reduced SCAP levels on synaptic transmission, we tested the synaptic efficacy of the Schaeffer collateral pathway between control and N-*Scap* (+/−) mice via paired-pulse facilitation and long-term potentiation (LTP). Paired-pulse facilitation was elicited with interpulse intervals of 50, 100, 150, and 200 ms, and then calculated as the paired-pulse ratio of field excitatory postsynaptic potential (fEPSP) amplitude elicited by the second to first test stimuli. The paired-pulse ratio was greatest when elicited with an interpulse interval of 50 ms and was significantly reduced in N-*Scap* (+/−) hippocampal slices (control: 1.73±0.08; *n* = 17; N-*Scap* (+/−): 1.45±0.04; *n* = 12; *p*<0.01) (Figure 4D and 4E), suggesting significant alterations in synapse plasticity in N-*Scap* (+/−) hippocampi. Further studies will be required to define the mechanism of this defect at the synaptic level.

We also tested the efficacy of neurotransmitter release following high frequency stimulation to determine if a robust and inducible LTP can be generated at CA1 synapses of N-*Scap* (+/−) hippocampal slices. High frequency stimulation (4×100 Hz) reliably induced a robust and reproducible LTP in hippocampal slices from both control and N-*Scap* (+/−) mice. However, the change in fEPSP slope was markedly reduced in N-*Scap* (+/−) slices (Figure 4F and 4G). The mean fEPSP slopes measured 10, 30, 60, and 120 min after LTP induction in control slices were 255±1, 189±4, 196±1, 188±1, and 183±1, respectively (*n* = 10) (Figure 4H). All corresponding slope values were significantly lower in slices from N-*Scap* (+/−) mice (198±3, 147±1, 139±2, 127±2, and 125±2, respectively; *n* = 8; *p*<0.001) (Figure 4H). Taken together, these findings indicate a significant impairment of synaptic transmission at CA1 neurons of the hippocampus in the brains of SCAP heterozygous knockout mice.

### N-*Scap* (+/−) Mice Exhibit Impairment in Recognition of Novel Objects and Altered Response to Stress

**Figure 5:**
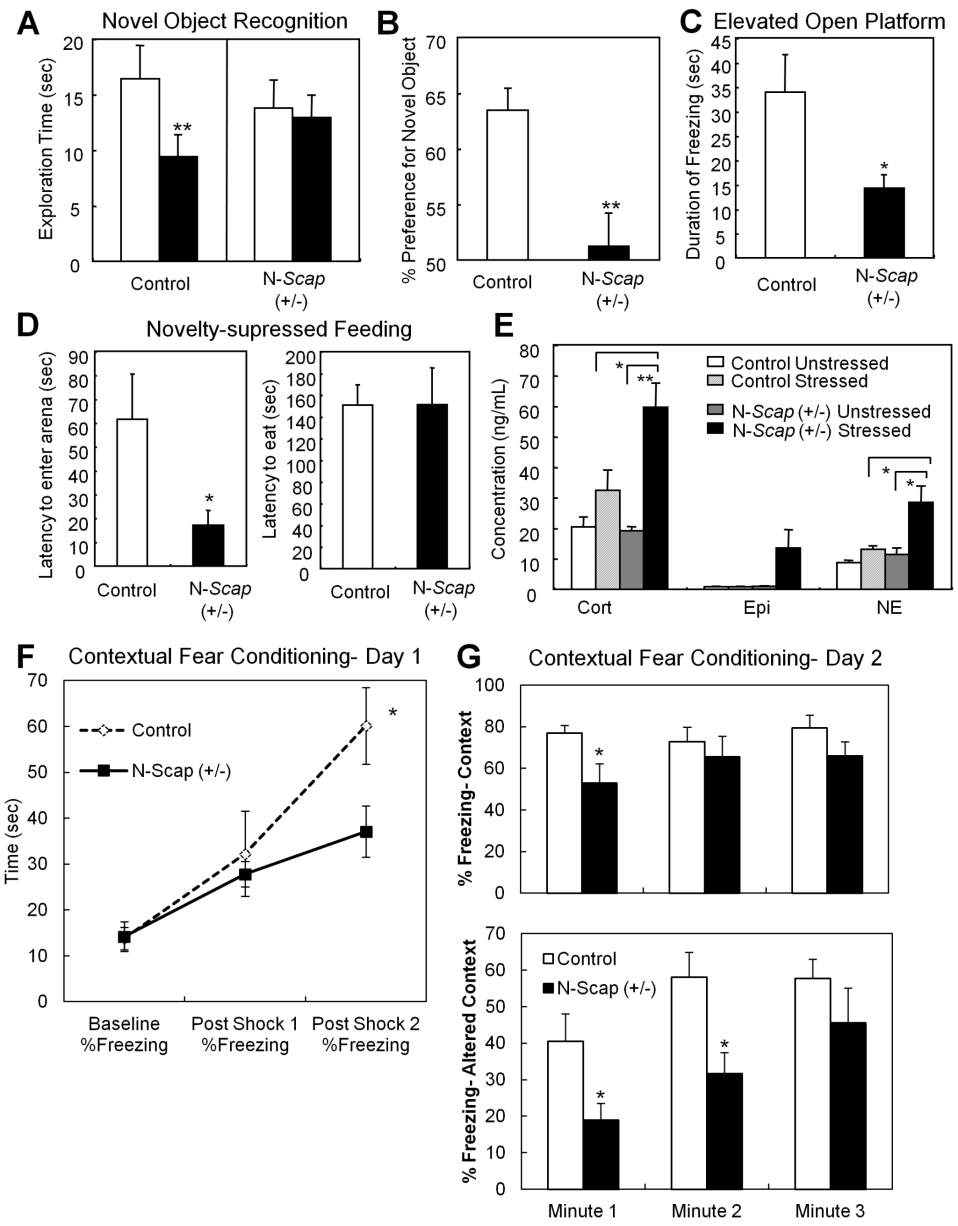
N-*Scap* (+/−) mice exhibit impaired cognitive function and abnormal behavior. (A) Novel object recognition test. Exploration time for a novel object (open bars) and a familiar object (closed bars) is indicated. **, *p*<0.01 by *t*-test. (B) Percentage of duration spent with the novel object in the same test. **, *p*<0.01 by *t*-test. (C) Elevated open platform test. Duration of freezing reaction on a platform during the 5-min test is indicated. *, *p*<0.05 by *t*-test. (D) Novelty-suppressed feeding test. Left panel shows latency to enter the novel arena. Right panel indicates latency to start eating the food pellets. *, *p*<0.05 by *t*-test. (E) Serum concentrations of stress-related hormones, corticosterone (Cort), epinephrine (Epi), and norepinephrine (NE) were measured under unstressed or stressed (after 1 h in isolation in a mouse transport container) conditions. (F) and (G) Contextual Fear Conditioning. The percentage of time in a freezing posture was measured at baseline and after shock 1 and 2 on day 1. On day 2, the percentage of time freezing was measured in both the same container from day 1 (context) and a new container (altered context).*, *p*<0.05; **, *p*<0.01.

Memory is impaired in patients with type 2 diabetes and to a lesser extent in patients with type 1 diabetes [13],[14]. To assess memory in our mouse model we used the novel object recognition test, which takes advantage of the natural bias of preference for novelty in rodents [15]. As expected, after a training session, control mice spent significantly more time exploring the novel object as compared to the training object (16.4±3 s versus 9.4±2 s, *p*<0.01), resulting in a 63% preference for the novel object (Figure 5A and 5B). By contrast, N-*Scap* (+/−) mice failed to demonstrate any significant preference for the novel object over the training object (13.8±2.5 s versus 12.9±2.1 s), spending almost identical amounts of time with each object (Figure 5A and 5B).

Diabetic patients have an increased prevalence of anxiety disorders [16],[17]. We used two different tests to assess anxiety in the N-*Scap* (+/−) mice. We performed the elevated open-platform test, which is used to assess psychological stress [18]. While controls spent 33.9±7.7 s in a frozen posture on the open platform, the N-*Scap* (+/−) mice exhibited a much shorter duration of freezing behavior (14.3±2.7 s, *p*<0.05) induced by this stress (Figure 5C). The novelty-suppressed feeding test assesses the effects of conflicting motivations of the drive to eat food after fasting and the fear of venturing into a novel arena of white paper on which the food pellets have been placed [19]. N-*Scap* (+/−) mice showed a markedly reduced latency to enter the arena compared to control mice, (17.1±6.4 versus 61.5±19 s, *p*<0.05) i.e., were more adventuresome or reckless in their behavior than controls (Figure 5D). Once they entered the novel area, however, there was no difference in the latency to start eating the food pellets (151.2±34.1 versus 150.7±18.8 s) (Figure 5D), demonstrating that hunger was not the driver of the reduced latency.

Interestingly, in unstressed conditions, circulating levels of the stress-related hormones corticosterone, epinephrine, and norepinephrine did not differ between control and N-*Scap* (+/−) mice. However, when the mice were isolated in individual transport containers for an hour, there was a robust elevation of stress hormone levels in N-*Scap* (+/−) mice compared with control mice with elevations in corticosterone (1.8-fold) and norepinephrine (2.2-fold) and a trend towards increased epinephrine (15-fold) (Figure 5E). The surprising increase in stress hormones in these mice despite an apparent decrease in anxiety behaviors may indicate an inappropriate reaction to stress rather than a true anxiolytic effect.

To further explore the possible relationship between behavior and the abnormal recordings found in the hippocampi of these mice we performed two behavioral tests which specifically use hippocampal function: contextual fear conditioning and the Morris water maze. In the contextual fear conditioning experiment N-*Scap* (+/−) mice showed impaired acquisition of the fear conditioning in response to a foot shock, as demonstrated by a decrease in freezing (Figure 5F). In addition, on the second day there was reduced freezing during the first minute in the context and the first and second minute in the altered context (Figure 5G), with later time points maintaining the same trend but losing statistical significance. This testing suggests impairments in hippocampal functioning, although we cannot rule out the possibility that there is some difference in pain thresholds between the mouse genotypes. We also performed a Morris water maze. We were unable to see significant differences between the two genotypes in this test though the N-*Scap* (+/−) mice showed a trend towards spending more time in the incorrect quadrants during the probe trials (Figure S4). Similar discordance between abnormal recordings in the hippocampus and abnormalities in the water maze have been previously observed in other models [20].

### Metabolic and Activity Characteristics of N-*Scap* (+/−) Mice

**Figure 6:**
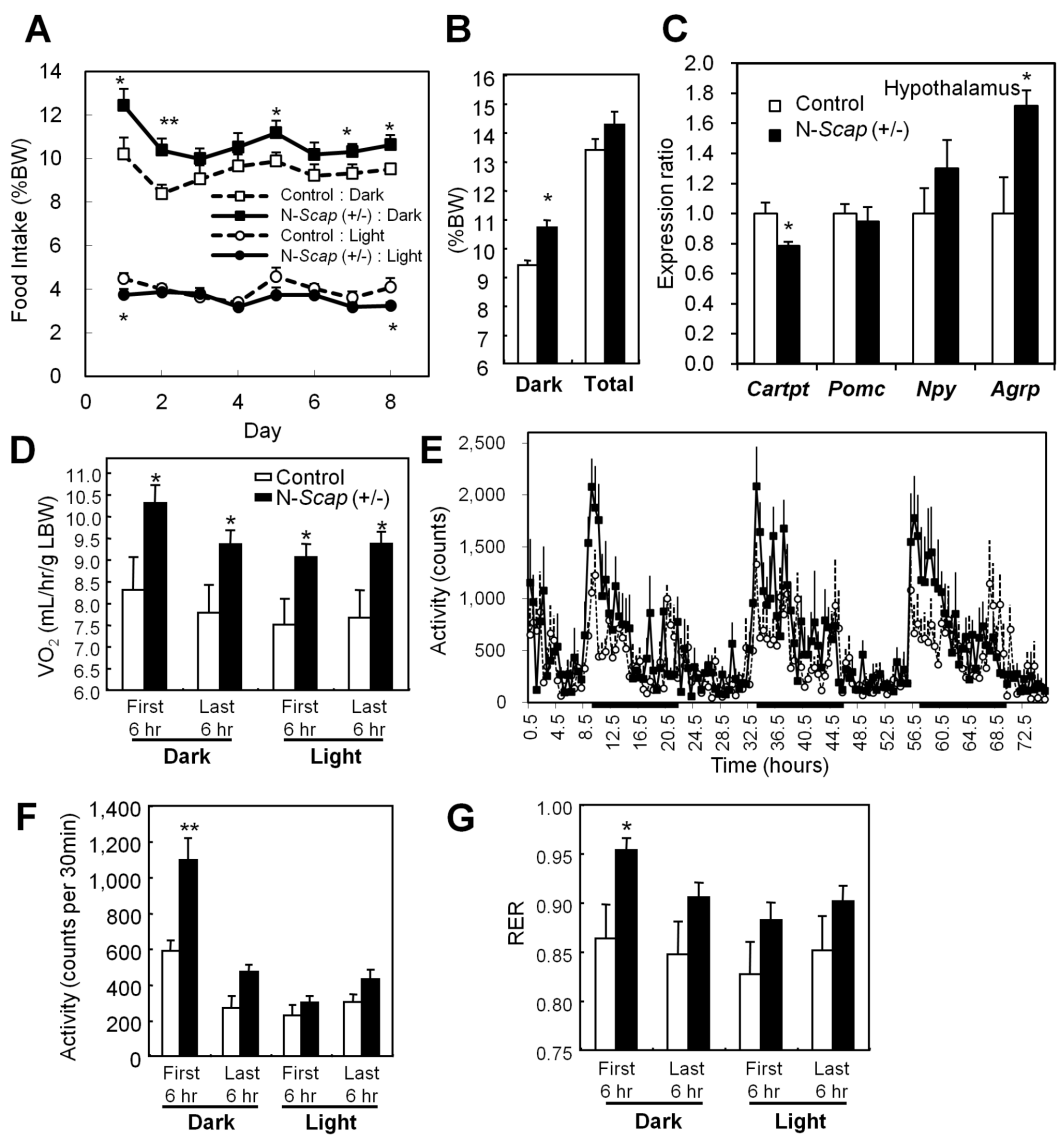
Feeding behavior and energy expenditure were altered in N-*Scap* (+/−) mice. (A) Food intake of the 12-wk-old control (*n* = 7) and N-*Scap* (+/−) mice (*n* = 7) were measured twice a day for 8 consecutive days. (B) Average food intake during light and dark phase. (C) Expression levels of appetite-related neuropeptide genes in the hypothalami as measured by real time PCR. (D–F) Oxygen consumption (D), locomotor activity (E), average of locomotor activity monitored for 3 d (F), and respiratory exchange ratio (RER) (G) were measured by CLAMS. (G) Average of locomotor activity monitored for 3 d in the CLAMS analysis. Control (*n* = 7, open bars) and N-*Scap* (+/−) mice (*n* = 7, closed bars) were compared. *, *p*<0.5; **, *p*<0.01 by *t*-test.

Finally, we evaluated feeding behavior and energy expenditure as measures of hypothalamic function in these mice. The N-*Scap* (+/−) mice consumed more food than the control mice, especially during the dark phase (Figure 6A and 6B). This phenotype is consistent with mice following SREBP-2 gene silencing in the hypothalamus shown in our previous study [9]. This correlates with mildly reduced expression of the anorexigenic neuropeptide CART (*Cartpt*), and increased expression of the orexigenic neuropeptide *Agrp* in the hypothalamus of the N-*Scap* (+/−) mice (Figure 6C). Energy expenditure, as represented by oxygen consumption in a metabolic cage, was about 20% higher in the N-*Scap* (+/−) mice than in control mice (Figure 6D). Consistent with this, locomotor activity of N-*Scap* (+/−) mice during this period indicated a significant, parallel increase (Figure 6E and 6F). The increase in energy expenditure compensated for the increase in food intake, resulting in similar body composition (Table S1). The respiratory exchange ratio (RER) was also high in the N-*Scap* (+/−) mice during the first 6 h of the dark cycle (Figure 6G), suggesting increased utilization of carbohydrate as a fuel.

## Discussion

In our previous study we showed that cholesterol synthesis is significantly impaired in multiple mouse models of diabetes due to a down-regulation of most of the genes in the cholesterol biosynthesis pathway [9]. In this study we show that this is due, at least in part, to a significant reduction of the sterol sensor protein SCAP in the brains of diabetic mice. Here we have used genetically modified mice with a defect in cholesterol synthesis to determine the consequences of decreased brain cholesterol, in the absence of the hyperglycemia and impaired insulin signaling found in diabetic mice. Homozygous deletion of *Scap* in the brain causes microcephaly, gliosis, and early postnatal lethality. Mice with a brain-specific *Scap* heterozygous knockout, on the other hand, closely mimic the decrease in sterol synthesis observed in the diabetic brain, and this leads to significant attenuation of synaptic transmission and cognitive dysfunction, even in the absence of any systemic metabolic derangement. Because *Scap* is knocked out during the prenatal period we cannot rule out a developmental contribution to the changes observed.

**Figure 7:**
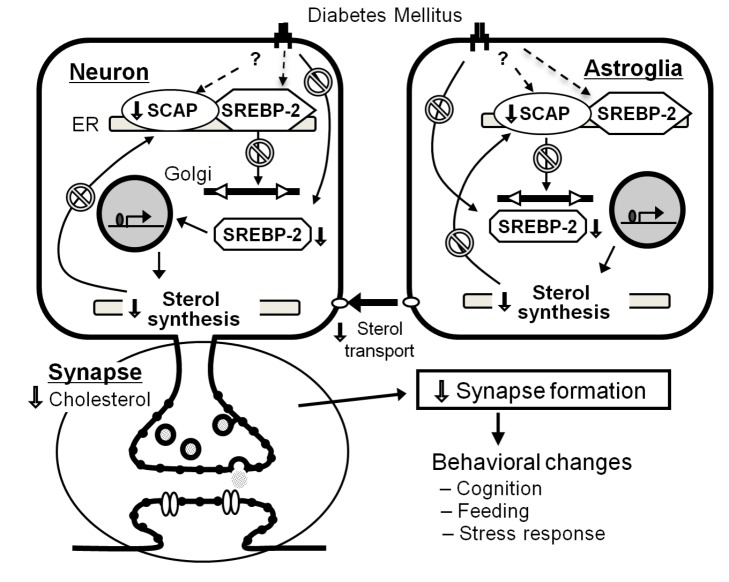
Proposed model for SCAP contribution to cognitive dysfunction and altered metabolism in diabetes. Diabetes reduces SCAP and mature SREBP-2 in the brain resulting in a reduction of cholesterol synthesis and cerebral decrease in synaptosomal cholesterol. This causes a decrease in normal synapse formation, altering synapse transmission, cerebral control of metabolism and various behaviors, including cognitive function.

Cholesterol comprises a significant component of the neuronal membrane and is thus critical for proper neuronal transmission. Reduction in cholesterol synthesis decreases the formation of synaptic contacts between neurons [9]. We show here that the reduction of cholesterol synthesis in the brains of N-*Scap* (+/−) mice produces adverse effects on neuronal transmission by decreasing the efficacy of synaptic transmission. Diabetic rodents exhibit impaired performance in the novel object recognition test and contextual fear conditioning test—functional measures of memory in rodents [21],[22]—and this is mimicked in the N-*Scap* (+/−) mouse. Impairment of LTP expression in the CA1 region of the hippocampus in diabetic animals has also been reported [23]–[25] and is also seen in the N-*Scap* (+/−) mouse. The impairment in LTP provides an electrophysiologic correlate for the impaired memory observed in the novel object recognition test and the contextual fear conditioning test. Coupled with our previous studies showing altered brain cholesterol metabolism in diabetes [9], the current results provide the first evidence indicating that failure in regulation of cholesterol homeostasis in the brain may contribute to the cognitive impairment seen in diabetes (see model in Figure 7). While the N-*Scap* heterozygous mouse model system does not allow for the precise dissection of the relative contributions of SREBP-2 processing and its effects on cholesterol synthesis versus SREBP-1 processing and its potential effects on fatty acid and triglyceride synthesis, it seems likely that SREBP-2 and cholesterol are more important. Although the knockout of SCAP produces changes to SREBP-1 processing, we find no defect in free fatty acid synthesis in the brains of N-*Scap* (+/−) mice (unpublished data), nor is there a decrease in triglyceride content in the brain. This is in contrast to a conditional knockout of SCAP in the liver, which causes decreases in serum free fatty acids and triglycerides of 46% and 53%, respectively, but only a 24% decrease in serum cholesterol [10]. This may be explained by the fact that while cholesterol is unable to cross the blood-brain barrier, fatty acids and glycerol, the building blocks of triglycerides, are able to pass. While fatty acid and glycerol transport across the blood-brain barrier may not fully compensate for all of the defects created by the decrease of SREBP-1 in the brain, it seems to significantly mitigate the effects given the normal to increased triglyceride levels observed in this manuscript. Nonetheless, the SREBP-1 pathway may have a greater impact in diabetes as there is a 15% decrease in fatty acid synthesis in brains of STZ diabetic mice (unpublished data). Further, there is also the possibility that components of the SREBP-2 pathway beyond what are explored here, such as isoprenoid production, may be contributing to the observed phenotype.

Exactly how lowering cellular cholesterol in the brain might affect brain function is unclear and likely multifactorial. Cholesterol depletion has been shown to retard or prevent clathrin-mediated endocytosis, including internalization of acetylcholine receptors [26] and block vesicle biogenesis in neurosecretory cells, consistent with a role for cholesterol in regulating membrane fluidity and the changes in curvature necessary for full vesicle formation [2],[27]. Moreover, cholesterol binds the synaptic vesicle protein synaptophysin and modulates interaction with the essential vesicular SNARE protein, synaptobrevin, to regulate exocytosis [28]. Knockdown of SREBP-2 in primary neuronal cultures produces decreases in expression of the synaptic vesicle marker VAMP2 [9]. Interestingly, deletion of low-density lipoprotein receptor-related protein 1 (LRP1) in forebrain neurons in mice leads to a decrease in brain cholesterol levels and memory loss with selective reduction of glutamate receptor subunits, which is partially rescued by restoring neuronal cholesterol [29]. These reports are consistent with the phenotypes of attenuated synapse transmission and cognitive impairment with decreased synaptosomal markers in N-*Scap* (+/−) mice.

Activation of glial cells, as demonstrated by increased GFAP staining, is a common feature of many types of neural insults including trauma, toxins, neurodegenerative triggers, or infection [30]. Multiple studies have reported increased GFAP expression or astrocyte content in cerebral cortices and hippocampi of diabetic animal models [21],[31],[32]; however, the mechanisms producing these changes have not been elucidated. The brains of N-*Scap* (−/−) mice show increased GFAP expression and gliosis. Whether this is a compensatory or reactive response induced by cholesterol deficiency in the brain remains to be determined.

Several neurodegenerative diseases have been associated with alterations in cholesterol metabolism. Reduction of cholesterol synthesis in astrocytes is thought to contribute to neurodegeneration in multiple models of Huntington disease [33]. Alzheimer disease has been associated with both diabetes and cholesterol metabolism [34]–[37]. The ε4 allele of ApoE, a cholesterol transport protein, is the only known risk factor, other than aging, for late onset Alzheimer disease [35]. On the other hand, studies examining the relationship of serum cholesterol levels in the elderly to dementia have differing conclusions, and several clinical trials in humans using the statin class of lipid lowering drugs have yielded conflicting results (reviewed in [36]). Part of this may reflect the broad range of human physiology. For example, in our previous study we showed a 3-fold range of expression of *Srebf2* and *Hmgcr* and a 2-fold range in synaptosomal cholesterol content in brains of 16 elderly humans with and without diabetes and dementia [9]. Based on our studies and others [36], one might predict that too much cholesterol, as well as too little, could have detrimental effects on neuronal function. However, because cholesterol in the brain is controlled independently of serum cholesterol, we do not currently have a clinically useful tool for assessing cholesterol levels in the human brain. Interestingly, a recent study used intranasal delivery of insulin to the brain as a therapy for human dementia patients with some improvement seen in memory and self care tasks [38]. The source of this benefit is unknown, but one of the responses to this therapy may be an increase in cholesterol synthesis in the brain.

Taken together with our previous work [9] we propose a model for how diabetes may affect cholesterol in the brain, leading to changes in behavior and brain function (Figure 7). In this model, diabetes mellitus reduces expression of SCAP in the brain, and this reduction of SCAP causes a defect in SREBP-2 processing, leading to a reduction in active SREBP-2. This leads to a down-regulation of genes involved in brain cholesterol synthesis, which leads to impaired synaptic transmission and abnormal cognitive function. These findings provide a novel view on the role of cholesterol regulation in the brain in diabetes, and open the possibility of therapeutic strategies for reversing the effects of diabetes on the brain and nervous system.

## Materials and Methods

### Animals

All animal studies followed the US National Institutes of Health guidelines and were approved by the Institutional Animal Care and Use Committees at the Joslin Diabetes Center and Beth Israel Deaconess Medical Center.

*Scap*-floxed mice [10], nestin-Cre mice [11], C57BL/6 mice, and *db/db* mice (C57Bl/Ks background) were purchased from the Jackson Laboratory. *Scap*-floxed mice with the *nestin-Cre* transgene were maintained on a C57BL/6×129Sv mixed genetic background; therefore for all studies littermates were used for analysis. For STZ-induced diabetes experiments, 7-wk-old C57BL/6 mice were treated with a single intraperitoneal injection (200 µg/g body weight) of STZ (Sigma). Some of the STZ diabetic mice were treated ICV with insulin. For these experiments 7-wk-old C57Bl/6 mice were placed in a stereotactic device under anesthesia, and a 26-gauge guide cannula (Plastics One Inc) was inserted into the right lateral cerebral ventricle (1.0 mm posterior, 1.0 mm lateral, and 2.0 mm ventral to the bregma). A dummy stylet was inserted into each cannula until used. After 1 wk of recovery, the mice received a single IP injection of STZ to induce diabetes. Twelve days later, the mice received three ICV injections of insulin (3 mU in 2 µl) or the same volume of PBS (9am, 7pm, and 9am the following day) through an internal cannula using a Hamilton microsyringe. Four hours after the last injection the hypothalami were collected. All mice were maintained on a 12-h light/12-h dark cycle and fed a standard mouse chow diet (LabDiet Mouse Diet 9 F, PMI Nutrition International). All mice used for experiments were male. For analysis of gene and protein expression, the brain was quickly removed under anesthesia with 2.5% Avertin (15 µl/g body weight, IP), placed on ice, and dissected into the hypothalamus and cerebral cortex using a mouse brain matrix (ASI Instruments Inc.).

### RNA Extraction and Gene Expression Analysis by Real-Time PCR

RNA from murine brain tissue was isolated using an RNeasy kit (Qiagen). As a template, 1 µg of total RNA was reverse-transcribed in 50 µl using a High Capacity cDNA Reverse Transcription kit (Applied Biosystems) according to the manufacturer's instructions. Three microliters of diluted (1∶4) reverse transcription reaction was amplified with specific primers (300 nM each) in a 25 µl PCR reaction with a SYBR Green PCR Master Mix (Applied Biosystems). Analysis of gene expression was done in the ABI PRISM 7000 sequence detector with initial denaturation at 95°C for 10 min followed by 40 PCR cycles, each cycle consisting of 95°C for 15 s and 60°C for 1 min, and SYBR green fluorescence emissions were monitored after each cycle. For each gene, mRNA expression was calculated relative to *Tbp* expression as an internal control.

### Immunohistochemistry

Brains were immersed in 4% paraformaldehyde (PFA) overnight at 4°C then embedded in paraffin, and 8 µm coronal sections were collected. Sections were deparaffinized and blocked with a Mouse Ig Blocking Reagent (M.O.M. Immunodetection kit, Vector Laboratories) containing avidin for 1 h, washed with PBS, treated with M.O.M diluent for 5 min, and incubated with a mouse monoclonal antibody recognizing GFAP (1∶500, Millipore) and biotin solution for 30 min at room temperature. After washing with PBS, the sections were incubated with a biotinylated anti-mouse IgG secondary antibody for 20 min. The samples were washed with PBS, treated with a streptavidin/peroxidase complex reagent, washed with PBS again, and stained with a VIP Substrate kit (Vector Laboratories). Hematoxylin was used for counter-staining.

### Immunoblotting

Nuclear and cytoplasmic extracts of brain tissue were prepared per the manufacturer's directions (NE-PER kit, Pierce). Whole tissue extracts were prepared using RIPA buffer containing 1% SDS and protease inhibitor cocktail (Sigma). Protein concentrations were measured using a BCA assay (Pierce). Immunoblotting was performed with antibodies against SCAP (Santa Cruz), β-tubulin, CREB, fatty acid synthase, acetyl-CoA carboxylase (Cell Signaling Technology), SQLE (ProteinTech), SREBP-1/SREBP-2 (gifts from Jay Horton and Guosheng Liang), STX1A and PSD95 (Abcam), and SYP and MBP (Millipore).

### Cholesterol Triglyceride Content

Brain tissue was homogenized in 50 mM NaCl. Lipid fraction was then extracted through multiple washes with a 2∶1 chloroform∶methanol solution. Samples were dried down with 10% triton-X 100/acetone. Cholesterol content was assayed by enzymatic assay (Wako Chemicals). Triglycerides were measured by colorimetric assay (Abnova).

### In Vivo Cholesterol Synthesis in the Brain

Each anesthetized animal was injected intraperitoneally with 50 mCi of [^3^H]water in 0.2 ml of PBS. One hour after injection (which is a time long enough to reflect endogenous rates of synthesis and short enough to avoid significant inter-organ redistribution [39]), blood was collected by retro-orbital puncture, and the [^3^H]water specific activity in the plasma was measured. The brain was removed, and the whole cerebrum (250–290 mg) was saponified with 2.5 ml of 2.5 M KOH (75°C, 2 h). Sterol-containing lipids were extracted with 10 ml hexane and 5 ml 80% ethanol. Cholesterol was isolated by thin layer chromatography (hexane∶diethyl ether∶glacial acetic acid = 80∶20∶1), and the incorporated tracer was measured by a scintillation counter. The synthesis rates were calculated as nmol of [^3^H]water incorporated into cholesterol per gram of tissue per hour.

### Electrophysiology

Transverse hippocampal slices (400 µm) were prepared from the brains of 4–6-wk-old male control or N-*Scap* (+/−) littermates that were submerged in an ice-cold high sucrose solution containing (in mM): 250 sucrose, 2.5 KCl, 1.24 NaH_2_PO_4_, 10 MgCl_2_, 10 glucose, 26 NaHCO_3_, 0.5 CaCl_2_ that was aerated with 95% O_2_/5% CO_2_. Slices were then maintained at 30°C in artificial cerebrospinal fluid (ACSF) containing (in mM): 124 NaCl, 2.5 KCl, 1.24 NaH_2_PO_4_, 1.3 MgCl_2_, 10 glucose, 26 NaHCO_3_, 2.5 CaCl_2_, and allowed to recover for at least 1 h before being transferred to the recording chamber for the start of experiments.

Voltage-clamp whole-cell recordings of mEPSC events at area CA1 neurons were recorded at a holding potential of −65 mV in the presence of 500 nM tetrodotoxin (TTX) using a glass microelectrode (9–10 MΩ) filled with an internal pipette solution containing (in mM): 137 K-gluconate, 2 KCl, 5 HEPES, 5 MgATP, 0.3 NaGTP, 10 creatine (290 mOsm/l [pH 7.4]).

fEPSPs were elicited by stimulating the Schaeffer collaterals with a concentric bipolar electrode (FHC) and recorded with an ACSF-filled glass microelectrode (1–2 MΩ) positioned in the stratum radiatum of area CA1. Baseline test stimuli were applied once per min at a test stimulation intensity (0.1 ms pulse width) adjusted to evoke fEPSP amplitudes that were 40% of maximal size. Paired-pulse stimulation was elicited by two consecutive test stimuli; the interpulse interval was varied from 50–200 ms, at 50 ms increments. LTP was induced by four trains of 100 Hz (1 s train duration) elicited 5 min apart. fEPSPs were not monitored between each high frequency train but were monitored with the test stimuli for up to 120 min after the induction of LTP.

All recordings were acquired with a Multiclamp 700B amplifier (Molecular Devices) via a Digidata 1440A (Molecular Devices) digitizer interface then recorded with pCLAMP 10.2 software. Offline analysis of mEPSCs and fEPSPs were performed with MiniAnalysis (Synaptosoft) and Clampfit 10.2 (Molecular Devices), respectively. Statistics and graphs were produced using Prism 5 (GraphPad Software). Statistical significance for mean comparisons was determined by the unpaired Student's *t* test at *p*<0.05. Data were represented as mean ± SEM, where appropriate, and *n* refers to the number of hippocampal slices, unless indicated otherwise.

### Novel Object Recognition Test

The novel object recognition test was performed as described previously [40] with slight modifications. Mice were maintained in group housing over the course of the experiment. Briefly, the mice were habituated for 1 h to a plastic cage box on the first day. The floor was covered with 1 cm of wood bedding. On the second day, a familiarization trial was performed for 5 min, allowing each mouse to explore the two identical objects (objects A and B) in the same box. The two objects were placed along the long axis of the box. Mice were filmed while exploring objects A and B and this was quantified to ensure that the mice did not show preference for the object on one side of the cage over the other. There was not a significant difference between exploration of object A and object B. Then, the mouse was removed from the trial box and placed in its home cage for 1 h. After each exposure, the objects were wiped with 70% ethanol to eliminate odor clues. One hour after the familiarization, each mouse was placed in the box with one of the old objects (object A) and a new one (object C). The position of object C was the same as object B in the familiarization trial, and the time to explore them was again 5 min. A mouse was considered to be engaging in exploratory behavior if the animal touched the object with its forepaw or nose or sniffed at the object within a distance of 1 cm. Activity was monitored and calculated from a timed video of the experimental field. In a separate cohort of mice both the old object (object A) and the new object (object C) were presented to mice for 30 s to determine if one object was more interesting to the mice than the other. There was no difference in exploratory behavior between the two objects when presented simultaneously.

### Novelty-Suppressed Feeding Test

The novelty-suppressed feeding test was performed as described previously [19] with slight modifications. Twenty-four hours before the test, food was removed from the cages. At the time of testing, a pair of food pellets (regular chow) were placed on a white paper disk positioned in the center of a trial box without wood bedding. An animal was placed in the corner. The latency to enter the arena and then to chew the pellets were recorded within a 5 min period. Activity was monitored and calculated from a timed video of the experimental field.

### Elevated Open Platform Test

The elevated open platform test was performed as described previously [18] with slight modifications. In brief, a transparent glass cylinder (12 cm diameter, 21 cm high) was placed upside-down and each mouse was positioned at the top (open platform). Freezing behavior was defined as no movement, excluding respiratory movement, while in a crouching posture. The duration of freezing was the total amount of time that the animal showed freezing. If the mouse slipped off the platform, it was immediately placed back on the platform and the experiment was continued. The mouse behavior on the elevated open platform was video recorded for 5 min.

### Morris Water Maze

During the acquisition phase mice were randomly dropped at one of four points; N, S, E, or W. A hidden platform was located in the southwest (SW) quadrant. Mice were given 1 min to find the hidden platform and latency was recorded. Mice were led to the hidden platform if they did not reach it within 1 min. Each mouse went through eight trials per day. Latency times leveled off on day 4. On day 5 the probe trial was conducted. The hidden platform was removed and the mouse was dropped off at the N drop point. Time spent in each quadrant was recorded over 1 min. Data was analyzed using TopScan software from Cleversys, Inc.

### Contextual Fear Conditioning

On day one each mouse was placed in a novel box for 2 min and freezing activity was recorded (baseline). At the end of the 2 min the mouse received a 0.5 mA shock for 2 s. Freezing time was again measured over 2 min (post shock 1). At the end of the 2 min a second 0.5 mA shock was delivered over 2 s and freezing was recorded for 1 min. The mouse was then returned to its home cage. On day 2 each mouse was placed back in the cage from day 1 and freezing was recorded for 3 min (context). The mice were then placed in an unfamiliar box and freezing was again measured for 3 min (altered context). Data was analyzed using TopScan software from Cleversys, Inc.

### Statistical Analysis

Data are expressed as the mean ± SEM. Statistical significance was calculated using an unpaired Student's *t*-test for comparison between two groups, and by an analysis of variance (ANOVA) for multigroup comparison. Fisher's PLSD was used for the Morris water maze and contextual fear conditioning.

## Supporting Information

Figure S1 N-Scap (−/−) mice express less GFAP in the brain. mRNA expression levels of *Gfap* (an astrocyte marker), *Emr1* (a microglia marker), and *Mtap2* (a neuron marker) in the brains of control, N-*Scap* (+/−), and N-*Scap* (−/−) mice. *, *p*<0.05; **, *p*<0.01 by *t*-test.
(TIF)

Figure S2 SREBP-1 target proteins are significantly reduced in N-*Scap* (−/−) mice. Western blots of whole cell lysates from P0 brains blotted for fatty acid synthase (FAS), acetyl-CoA carboxylase (ACC) or β-tubulin.
(TIF)

Figure S3 There is no change in Cyp46a1 expression in N-*Scap* (+/−) or N- *Scap* (−/−) mice. mRNA expression levels of Cyp46a1 in (A) cortex and hypothalamus from 5-wk-old mice and (B) whole brain from P0 mice. None of the comparisons are significant.
(TIF)

Figure S4 There is no difference between control and N-*Scap* (+/−) in the Morris water maze. Mice were trained to the southwest (SW) quadrant. Distance and time spent in each quadrant was recorded for the probe trial. Control mice spent statistically more time and swam a greater distance in the SW quadrant than all others (**). The N-*Scap* (+/−) mice spent more time and swam a greater distance in the SW quadrant than the NE and SE quadrants but not the NW quadrant (*).
(TIF)

Table S1 Body composition of N-*Scap* (+/−) mice is similar to control. Body weight, lean mass, and fat mass were determined from dual-energy X-ray absorptiometry (DEXA) scanning. Femur lengths were measured from images generated by the DEXA scans and scaled by 0.65 owing to enlargement of the images from the DEXA scanning. Femur length is smaller in the N-*Scap* (+/−) mice but body composition is unchanged.
(TIF)

## Abbreviations

fEPSP: field excitatory postsynaptic potential
ICV: intracerebroventricular
LTP: long term potentiation
mEPSC: miniature excitatory postsynaptic current
STZ: streptozotocin
