# Supplementary figures and images for: Reduction of the Cholesterol Sensor SCAP in the Brains of Mice Causes Impaired Synaptic Transmission and Altered Cognitive Function
Source: PLoS Biol. 2013 Apr 9;11(4):e1001532. doi: 10.1371/journal.pbio.1001532 (PMC3621654; doi:10.1371/journal.pbio.1001532)

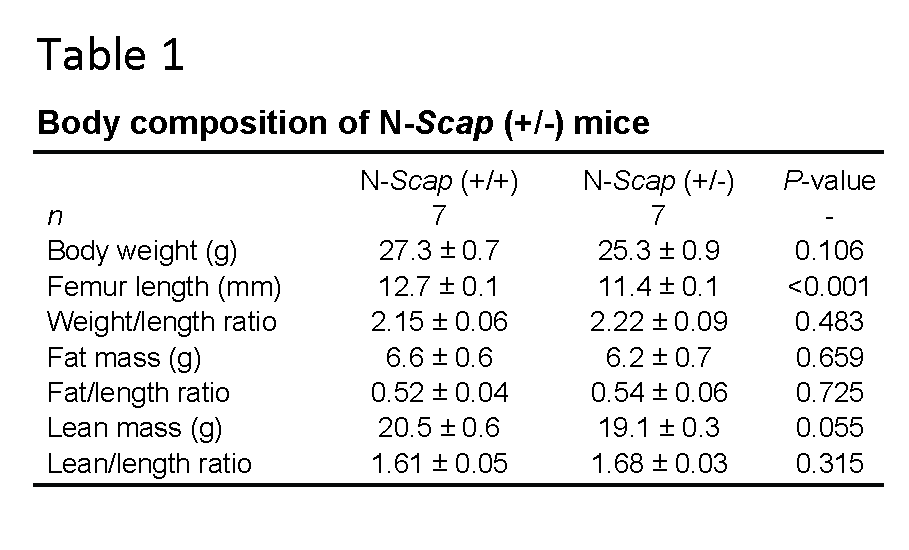

Supplement: Table S1 — Body composition of N-Scap (+/−) mice is similar to control. Body weight, lean mass, and fat mass were determined from dual-energy X-ray absorptiometry (DEXA) scanning. Femur lengths were measured from images generated by the DEXA scans and scaled by 0.65 owing to enlargement of the images from the DEXA scanning. Femur length is smaller in the N-Scap (+/−) mice but body composition is unchanged. (TIF) [file pbio.1001532.s005.tif]
